# Characterizing Health Care Delays and Interruptions in the United States During the COVID-19 Pandemic: Internet-Based, Cross-sectional Survey Study

**DOI:** 10.2196/25446

**Published:** 2021-05-19

**Authors:** Elizabeth Lerner Papautsky, Dylan R Rice, Hana Ghoneima, Anna Laura W McKowen, Nicholas Anderson, Angie R Wootton, Cindy Veldhuis

**Affiliations:** 1 Department of Biomedical & Health Information Sciences College of Applied Health Sciences University of Illinois at Chicago Chicago, IL United States; 2 Department of Neurology Massachusetts General Hospital Boston, MA United States; 3 Department of Counseling & Clinical Psychology Teachers College Columbia University New York, NY United States; 4 School of Social Welfare University of California Berkeley Berkeley, CA United States

**Keywords:** COVID-19, health care delays, internet survey, preventive care, delay, interruption, lockdown, precaution, prevention, social media, survey

## Abstract

**Background:**

The COVID-19 pandemic has broader geographic spread and potentially longer lasting effects than those of previous disasters. Necessary preventive precautions for the transmission of COVID-19 has resulted in delays for in-person health care services, especially at the outset of the pandemic.

**Objective:**

Among a US sample, we examined the rates of delays (defined as cancellations and postponements) in health care at the outset of the pandemic and characterized the reasons for such delays.

**Methods:**

As part of an internet-based survey that was distributed on social media in April 2020, we asked a US–based convenience sample of 2570 participants about delays in their health care resulting from the COVID-19 pandemic. Participant demographics and self-reported worries about general health and the COVID-19 pandemic were explored as potent determinants of health care delays. In addition to all delays, we focused on the following three main types of delays, which were the primary outcomes in this study: dental, preventive, and diagnostic care delays. For each outcome, we used bivariate statistical tests (*t* tests and chi-square tests) and multiple logistic regression models to determine which factors were associated with health care delays.

**Results:**

The top reported barrier to receiving health care was the fear of SARS-CoV-2 infection (126/374, 33.6%). Almost half (1227/2570, 47.7%) of the participants reported experiencing health care delays. Among those who experienced health care delays and further clarified the type of delay they experienced (921/1227, 75.1%), the top three reported types of care that were affected by delays included dental (351/921, 38.1%), preventive (269/921, 29.2%), and diagnostic (151/921, 16.4%) care. The logistic regression models showed that age (*P*<.001), gender identity (*P*<.001), education (*P*=.007), and self-reported worry about general health (*P*<.001) were significantly associated with experiencing health care delays. Self-reported worry about general health was negatively related to experiencing delays in dental care. However, this predictor was positively associated with delays in diagnostic testing based on the logistic regression model. Additionally, age was positively associated with delays in diagnostic testing. No factors remained significant in the multiple logistic regression for delays in preventive care, and although there was trend between race and delays (people of color experienced fewer delays than White participants), it was not significant (*P*=.06).

**Conclusions:**

The lessons learned from the initial surge of COVID-19 cases can inform systemic mitigation strategies for potential future disruptions. This study addresses the demand side of health care delays by exploring the determinants of such delays. More research on health care delays during the pandemic is needed, including research on their short- and long-term impacts on patient-level outcomes such as mortality, morbidity, mental health, people’s quality of life, and the experience of pain.

## Introduction

Times of disaster have often been characterized by a lack of access to health care, treatment delays or interruptions, and medication shortages [1-4]. The COVID-19 pandemic has broader geographic spread and potentially longer lasting effects than those of previous disasters. Further, the care of patients with COVID-19 has overburdened health care systems, and seeking health care may put patients at risk of exposure to SARS-CoV-2. Implementing necessary infection prevention precautions for the transmission of SARS-CoV-2 (the virus that causes COVID-19) and allocating resources (eg, personal protective equipment, beds, and personnel) in anticipation of or in response to a surge of COVID-19 cases has resulted in delays in and cancellations of in-person health care services, especially at the outset of the pandemic. These delays may affect a large proportion of patients. For example, in a recent study about health care delays at the outset of the pandemic among breast cancer survivors (April 2 to April 27, 2020; N=609), 44% of participants reported breast cancer treatment delays [5]. The consequences can be significant for individuals with conditions that require timely intervention, such as cancer, other chronic health conditions, and mental health concerns. The deleterious effects of postponing preventive, diagnostic, and dental health care may include the delayed or missed diagnosis of life-threatening illnesses such as cancer, the exacerbation of illnesses, and even death. Given the potential implications for the morbidity and mortality of delaying or not accessing health care, it is important to document delays and interruptions in health care access during the COVID-19 pandemic to inform appropriate intervention efforts.

Of great interest and concern are delays in preventive and diagnostic care, particularly delays in such care for older adults. Before the pandemic, approximately 50% of all adults received the recommended preventive care in the United States [6-8]. Thus, people with pre-existing and emerging conditions may not be receiving needed health care management—a problem that was not introduced but rather amplified by the pandemic. Further, as preventive and diagnostic care are the first line of defense for diagnosing life-threatening illness, delays and interruptions may result in higher mortality and morbidity. This is of particular concern for older adults, given that age is the main risk factor for cardiovascular disease, cancer, and neurodegenerative conditions [9], and such conditions may be exacerbated by stress and isolation during the pandemic. Emerging studies on delayed and interrupted care have focused on specialties such as head and neck malignancies [10], urologic surgeries [11], and heart failure [12]. Ding and colleagues [13] documented the potentially fatal consequences of care delays for COVID-19–negative patients requiring in-person medical care for timely diagnosis. They described five pediatric patient cases with poor clinical outcomes resulting from delays in cancer diagnosis. These poor outcomes unfortunately occurred despite the fact that several patients interacted with primary care providers through telehealth visits prior to presenting at the hospital. Due to the effects of the COVID-19 pandemic on people’s access to health care, the oncology community anticipates an uptick in the number of cancer diagnoses and diagnoses at higher cancer stages and an increase in cancer mortality rates overall [14]—a devastating and sobering concern. Research with a focus on older adults is needed to characterize the near- and long-term impacts of delayed and interrupted care on health and inform effective and person-centered solutions to protect severely ill but treatable patients.

To address a gap in our understanding of the effects of the pandemic on health care access, we examined the rates of delays in health care among a US convenience sample at the outset of the pandemic, with a specific focus on preventive care. Our study reflects Americans’ experiences with the demand for health care. We also characterized the reasons for such delays. As we navigate the pandemic and the potential resurgences of infections, our findings can inform systems-level strategies for future public health emergencies to mitigate delays, improve effective communications for addressing uncertainty, and promote mental health interventions.

## Methods

### Study Design

In April 5, 2020, we launched an internet-based survey study examining the impact of the COVID-19 pandemic on mental health and well-being. Participants were invited to take part in a study that focused on “how the pandemic is affecting you” through social media (ie, Twitter and Facebook), listservs, social networks, websites (eg, Buzzfeed), and the research match website at Columbia University. Information about this study was included in the beginning of the survey; participants indicated consent by proceeding to the survey. The survey comprised multiple validated instruments as well as items that were developed to help us understand experiences that were specific to the COVID-19 pandemic. The mean completion time for the full survey was approximately 35 minutes. As this study was not funded, participants were not offered compensation. At the culmination of the survey, participants were provided with a list of resources (eg, crisis hotlines) in case they experienced distress. Participants were also asked to provide their email addresses if they consented to being recontacted for future follow-ups. Participants who provided their email addresses were surveyed again between April 26 and May 5, 2020. All study procedures were reviewed and approved by the Columbia University Institutional Review Board.

### Measures

Participants were asked about any interruptions they experienced to their health care in the following item, which was developed specifically for this study: “Have you experienced any delays or interruptions in your healthcare (e.g. cancelled or delayed appointments, tests, procedures) during the coronavirus outbreak?” Response options for this item were dichotomous (“yes” or “no”), and participants who responded affirmatively were asked to elaborate with an open-ended, text-based answer. Responses ranged from no delayed care to multiple types of delayed care. Some participants included more detailed responses.

### Data Coding and Analysis

To characterize and code the types of delays, we used Microsoft Excel. Three researchers (ELP, DR, and HG) coded participants’ responses to the open-ended item about the types of health care delays and interruptions they experienced [15,16]. The coding process was as follows. First, one researcher (DR) reviewed a set of 25 randomly selected responses and developed a draft codebook, which primarily focused on the types of care affected. Second, the three researchers collaboratively reviewed the codebook draft and planned the logistics of the coding process. Each researcher coded the same 50 responses, identified coding uncertainties, and noted potential codebook edits. To establish interrater reliability, the three researchers discussed coding discrepancies until they were resolved with 100% agreement among all three researchers. The codebook was edited to clarify codes, add additional codes, and remove codes. Third, each researcher coded a third of the remaining responses. Any uncertainties were resolved by communication and discussion with the other two researchers. Finally, upon completion, researchers debriefed to further refine the codebook (eg, adding a dermatology code based on its frequency of appearance in the data set). All codes (n=2776) were integrated into a single spreadsheet.

### Statistical Analysis

Basic descriptive statistics were used to describe the research sample. We conducted two sets of inferential procedures to determine which factors were associated with self-reported health care delays, with a focus on three outcome variables. First, we examined the determinants of experiencing any health care delays due to the pandemic (dichotomous variable; “yes” or “no”). Among those who experienced delays, we were particularly interested in the following three types of delays: dental, preventive, and diagnostic care delays. For each dependent variable, bivariate analyses were conducted to describe the associations of the outcomes with demographic characteristics or self-reported levels of worry about their general health and COVID-19. Independent samples *t* tests were used for continuous independent variables, whereas chi-square tests were used for categorical variables. All independent variables were then entered into a multiple logistic regression model; R (The R Foundation) [17] was used for the statistical inference procedures.

## Results

The analytic sample for this study included 2570 participants (accounting for missing data in the delays question) from across all US states who completed the survey between April 5, 2020, and May 5, 2020. Participants’ ages ranged from 18 to 84 years (mean 37.3 years, SD 12.6 years). The majority of participants were non-Hispanic White (2464/2570, 95.9%), were cisgender (ie, gender identity is consistent with the sex assigned at birth) women (2456/2570, 95.6%), were heterosexual (1680/2570, 65.4%), and had at least a Bachelor’s degree (1259/2570, 50%). Table 1 shows a summary of demographics.

**Table 1:** Sample characteristics (N=2570).

Variable			Value
**Numeric variables, mean (SD)**			
	Age (years)		37.31 (12.63)
	Distance to hospital (miles)		2.30 (0.95)
	Health-related worry (scale of 0-100)		52.27 (26.71)
	COVID-19–related worry (scale of 0-100)		74.93 (20.54)
**Categorical variables, n (%)**			
	**Gender**		
		Cisgender women	2456 (95.6)
		Cisgender men	324 (12.6)
		Transgender or nonbinary individuals	118 (5.6)
	**Sexual identity**		
		Heterosexual	1680 (65.4)
		Sexual minority	1202 (46.8)
	**Race and ethnicity**		
		White	2464 (95.9)
		Black	48 (1.9)
		Latina, Latino, Latinx, or Hispanic	174 (6.8)
		American Indian or Alaska Native	0 (0)
		Asian or Pacific Islander	199 (7.7)
		Biracial	7 (<1)
	**Education**		
		High school or less	57 (2.2)
		Less than a Bachelor's degree	419 (16.3)
		Bachelor's degree	783 (30.5)
		Enrolled in graduate school	287 (11.2)
		Graduate or professional school	894 (34.8)
		Doctorate degree (eg, MD, PhD, DrPH)	449 (17.5)

**Figure 1:**
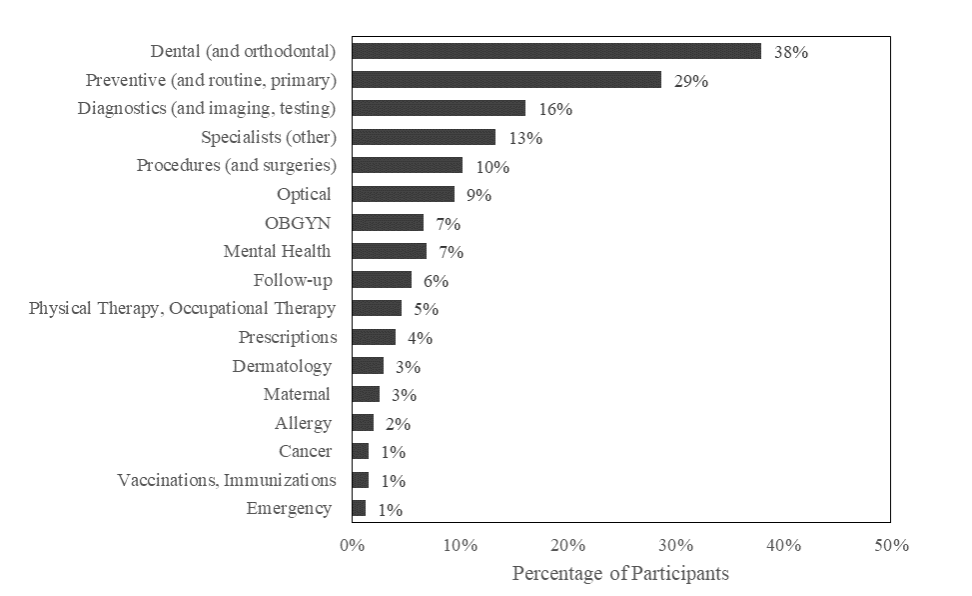
Types of health care impacted by the COVID-19 pandemic (n=921). Proportions do not sum to 100% because participants may have listed >1 type of care. OGBYN: obstetrics and gynecology.

Almost half (1227/2570, 47.8%) of all participants reported experiencing delays or interruptions in their health care. Of these, 8.3% (102/1227) reported switching their care to telemedicine, and 12.6% (154/1227) reported experiencing health care access issues (eg, barriers associated with insurance coverage). Most participants (921/1227, 75.1%) who experienced delays specified the types of affected care. Some individuals solely responded with phrases such as “doctor’s appointment,” which resulted in no associated code due to the lack of the specificity of the response. Among those that specified the type of care, the three most commonly reported types of care affected included dental (351/921, 38.1%), preventive (269/921, 29.2%), and diagnostics (151/921, 16.4%) care (Figure 1). Emergency care was the least commonly reported type of care affected (12/921, 1.3%) among this sample, followed by vaccinations (14/921, 1.5%) and cancer care (14/921, 1.5%). Further, 10.2% (94/921) of the participants who responded to this item reported disruptions to the health care of others (eg, child, spouse, and parent; not included in Figure 1).

In Table 2, we present the reported barriers to receiving health care services, which were based on follow-up questions that were sent to individuals who indicated an interest in participating in follow-up questionnaires. The top 3 barriers to care—each reported by about one-third of participants—included a fear of SARS-CoV-2 infection (126/374, 33.7%), provider discouragement (122/374, 32.6%), and the feeling that their health care concerns were not as important as others’ concerns (118/374, 31.6%).

**Table 2:** Barriers to receiving health care during the COVID-19 pandemic (n=1025). Responses to the following question are presented: “Since the start of the coronavirus pandemic, have you needed to get healthcare but haven't gotten it? Why?”

Responses	Value, n (%)^a^
No	651 (63.5)
Yes	374 (36.5)
I was afraid of getting infected	126 (33.7)
My health care provider discouraged me from coming in	122 (32.6)
I felt like my concern/need wasn’t as important as other people's	118 (31.6)
My health care provider is unavailable	108 (28.9)
I felt like my symptoms weren't severe enough	89 (23.8)
I have no health insurance	15 (4)
I cannot afford my copay or deductible	14 (3.7)
Other	60 (16)

^a^Proportions do not sum to 100% because participants may have listed >1 reason.

### Determinants of Patient-Reported Health Care Delays

Table 3 shows the associations between the dependent variable “any health care delays” and independent variables as a series of separate bivariate tests. Several demographic factors were associated with reporting any health care delays resulting from the pandemic. On average, older age, cisgender women, White individuals, higher levels of education, higher levels of self-reported worries about general health, and higher levels of self-reported worries about COVID-19 were all associated with experiencing health care delays. We then entered all independent variables, regardless of their significance at the bivariate level, into a multiple logistic regression model. The results from the logistic regression models indicated that age (*P*<.001), gender identity (*P*<.001), race (*P*=.005), education (*P*=.007), and the degree of worry about COVID-19 (*P*<.001) and general health (*P*<.001) were all significantly associated with experiencing health care delays due to the pandemic. The largest effect was observed for gender; the odds of patient-reported health care delays for cisgender men were 0.60 (95% CI 0.46-0.78) times that of cisgender women.

**Table 3:** Bivariate analyses^a^ of the reasons for experiencing health care delays resulting from the COVID-19 pandemic (N=2570).

Independent variables		Yes	No	*P* value
Age (years), mean (SD)		38.9 (13.1)	36.0 (12.1)	<.001
**Gender identity, %^b^**				
	Cisgender women	87.2	83.8	<.001
	Cisgender men	8.3	13.4	—^c^
	Transgender or nonbinary individuals	4.6	3.8	—
**Race, %^b^**				
	White	87.8	83.9	.005
	People of color	12.2	16.1	—
**Sexual identity, %^b^**				
	Heterosexual	60	57.6	.22
	Sexual minority	40	42.4	—
**Education, %^b^**				
	Less than a Bachelor’s degree	17	14.6	.007
	Bachelor’s degree or some graduate school	38.9	35.3	—
	Graduate or doctorate degree	44.1	50.2	—
Distance to nearest hospital (miles), mean (SD)		2.3 (1.0)	2.3 (0.9)	.15
Worry about general health (scale of 0-100), mean (SD)		54.5 (27.0)	50.1 (26.2)	<.001
Worry about COVID-19 (scale of 0-100), mean (SD)		76.3 (20.4)	73.6 (20.6)	<.001

^a^Bivariate tests are used to determine whether each independent variable is related to each dependent variable. Continuous independent variables are used in an independent samples *t* test to identify a significant association, while categorical independent variables are used in a chi-square test of association.

^b^Percentages are based on column totals.

^c^Not available.

We also examined delays in specific types of health care—dental, preventive, and diagnostics care. Both the bivariate tests (Table 4) and the multiple logistic regression model (Table 5) showed that the only significant independent variable that was associated with dental delays was self-reported worries about general health. Increased levels of worry were associated with fewer delays in dental care (*P*=.002). Our bivariate analyses identified two factors that were associated with experiencing delays in diagnostics (Table 4)—age and self-reported worry about general health. In the multiple logistic regression model, both factors remained significant at the .05 level of confidence. Age had a larger effect on experiencing delays in diagnostics. Every 10-year increase in age increased the odds of experiencing delays in diagnostics by a multiple of 1.36. The bivariate tests of association for delays in preventive care showed that age (*P*=.03) and race and ethnicity (*P*=.02) were significant. Neither of these factors remained significant in the multiple logistic regression model, and although there was trend between race and delays (people of color experienced fewer delays than White participants), it was not significant (*P*=.06).

**Table 4:** Bivariate analyses of the reasons for experiencing health care delays resulting from the COVID-19 pandemic among those who reported experiencing any health care delays (N=921).

Independent variables		Dependent variables																
		Delays in dental care					Delays in diagnostics							Delays in preventive care				
		Yes	No	*P* value		Yes		No		*P* value		Yes			No		*P* value	
Age (years), mean (SD)		39.3 (13.3)	39.3 (13.3)	.50		43.6 (13.3)		38.8 (13.1)		<.001		41.1 (13.3)			38.9 (13.0)		.03	
**Gender, %**					.94						.36							.24
	Cisgender women	86.6	87			88.1		86.6				89.2			85.9			
	Cisgender men	8.8	8.3			6		9				7.8			8.7			
	Transgender or nonbinary individuals	4.6	4.7			6		4.4				3			5.4			
**Race, %**					.99						.38							.02
	White	88.9	89.1			86.7		89.5				92.9			87.4			
	People of color	11.1	10.9			13.3		10.5				7.1			12.6			
**Sexual identity, %**					.22						.99							.07
	Heterosexual	61.1	56.9			58.3		58.5				63.2			56.5			
	Sexual minority	38.9	43.1			41.7		41.5				36.8			43.5			
**Education, %**					.89						.60							.09
	Less than a Bachelor’s degree	13.7	14.7			14.6		14.3				11.9			15.3			
	Bachelor’s degree or some graduate school	35.9	34.9			31.8		36				32.3			36.5			
	Graduate or doctorate degree	50.4	50.4			53.6		49.7				55.8			48.2			
Distance miles to nearest hospital (miles), mean (SD)		2.3 (0.9)	2.4 (1.0)	.25		2.3 (1.0)		2.3 (1.0)		.87		2.3 (0.9)			2.3 (1.0)		.73	
Worry about general health (scale of 0-100), mean (SD)		50.5 (27.2)	56.1 (26.4)	.002		60.2 (26.0)		52.8 (26.8)		.001		54.3 (25.2)			53.8 (27.5)		.81	
Worry about COVID (scale of 0-100), mean (SD)		75.8 (20.6)	77.1 (20.3)	.38		79.0 (19.3)		76.1 (20.6)		.11		77.2 (19.2)			76.3 (20.9)		.54	

**Table 5:** Multiple logistic regression of reasons for experiencing health care delays resulting from the COVID-19 pandemic.

Independent variables^a^		Dependent variables^b^							
		Any delays		Delays in dental care		Delays in diagnostics		Delays in preventive care	
		*P* value	95% CI	*P* value	95% CI	*P* value	95% CI	*P* value	95% CI
Age (years; 10-year increments)		<.001	1.12-1.28	.25	0.95-1.19	<.001	1.15-1.53	.18	0.96-1.22
**Gender**									
	Cisgender men	<.001	0.46-0.78	.93	0.60-1.59	.17	0.27-1.20	.60	0.50-1.46
	Transgender or nonbinary individuals	.36	0.80-1.81	.75	0.56-2.16	.48	0.55-3.03	.39	0.29-1.52
**Race**									
	People of color	.07	0.64-1.02	.70	0.70-1.68	.10	0.89-2.69	.05	0.34-0.99
**Sexual identity**									
	Sexual minority	.01	1.04-1.46	.28	0.63-1.14	.31	0.82-1.81	.26	0.61-1.14
**Education**									
	Bachelor’s degree or some graduate school	.43	0.87-1.40	.63	0.72-1.71	.87	0.60-1.90	.32	0.79-2.09
	Graduate or doctorate degree	.02	1.03-1.64	.85	0.69-1.57	.58	0.69-2.03	.07	0.96-2.39
Distance (miles) to nearest hospital		.34	0.96-1.13	.31	0.81-1.07	.68	0.80-1.16	.70	0.89-1.19
Worry about general health (10-point increments)		.01	1.01-1.08	.002	0.86-0.97	.02	1.01-1.19	.95	0.94-1.06
Worry about COVID (10-point inccrements)		.88	0.96-1.05	.35	0.96-1.12	.62	0.88-1.09	.72	0.93-1.10
Intercept		<.001	0.17-0.44	.41	0.31-1.62	<.001	0.01-0.12	<.001	0.09-0.53

^a^The reference group for all categorical variables are as follows: cisgender woman (gender), White (race), heterosexual (sexual identity), and less than Bachelor’s degree (education).

^b^There were 2570 observations for any delays, 907 observations for delays in dental care, 907 observations for delays in diagnostics, and 907 observations for delays in preventive care.

## Discussion

### Principal Findings

In June 2020, Dr Lasic, an interventional cardiologist at Jamaica Hospital Medical Center and Lenox Hill Hospital in New York, made the following prediction: “I think the toll on non-COVID patients will be much greater than COVID deaths” [18]. To our knowledge, ours is the only study that aimed at understanding the toll on patients without COVID-19 by describing rates of multiple types of health care delays as well as characterizing people who were the most affected by these delays during the beginning of the pandemic. In June 2020, under dynamic circumstances, researchers found that 32% and 12% of adults deliberately delayed or avoided routine care and emergency care, respectively [19]. Our findings suggest that a not insignificant proportion of the US populace may be experiencing delayed or interrupted care as a result of the pandemic, which may lead to decrements in health over the long term. Across all types of delays, older people, people with higher levels of education, and people who were more worried about their health were more likely to report delays or interruptions in their care. Men and people of color were less likely to report delays or interruptions.

Our findings highlight that dental care appears to be the most impacted (reported by over one-third of participants; 351/921, 38.1%). This is unsurprising, given that many dental offices closed at the outset of the pandemic due to concerns of patient and provider safety. However, studies have shown a relationship between dental health and heart disease, between dental health and diabetes, and between dental health and prenatal outcomes [20-22]. This is suggestive of the potential, downstream, deleterious health implications of delayed or interrupted dental care. Dentists are often also on the frontlines of identifying child abuse [23] and intimate partner violence [24], of which both have reportedly increased in incidence during the pandemic [25-27]. Further, a recent study conducted in Qatar has suggested that routine oral care is associated with a reduction in the risk of COVID-19 complications (eg, hospitalization and ventilation) [28]. Several months into the pandemic (July 2020), the American Dental Association released a policy declaring that dentistry was essential care (ie, care that is integral to systemic health) [29]. Of great concern are our findings associated with the frequency of reported delays in preventive and diagnostic care, particularly delays in such care for older adults. The implications of this finding are described in the *Introduction* section.

The lessons learned from the initial surge of COVID-19 cases can help mitigate potential future disruptions. Mitigation strategies for future disruptions include effective patient prioritization and triage [30-34]. For instance, Medically Necessary Time-Sensitive Prioritization is a scoring system for surgical triage that integrates multiple factors associated with the environment, patient risk, and ethics that are intended to be generalizable across hospital settings [35]. Additionally, the Centers for Medicare & Medicaid Services have released guidelines for the provision of non–COVID-19 care [36-38], However, considerations should also include patient life factors (eg, family support and transportation) as well as systems factors at the hospital (eg, visitation policy), local and state (eg, infection control mandates), or national levels (eg, policies). Using systems thinking perspectives is necessary for addressing complex problems [39]. Cancer care providers have also recommended balancing delays against COVID-19 risk, practicing effective social distancing, and managing the appropriate allocation of resources [32]. Key to reducing delays among patients is the relaying of accessible, clear, and actionable messaging by health care systems about how and when to safely access health care.

More research on health care delays and interruptions during the pandemic is needed. Further investigation is needed into the differences between the care of chronic illness and emergent conditions. For instance, the first pandemic surge resulted in a decline in hospitalization rates and lengths of hospital stay associated with acute cardiovascular conditions as well as other common conditions, such as acute appendicitis, bone fractures, cancer, and live births. Given the evidence on differences based on sex [40], race and ethnicity [41], sexual and gender identity [42,43], and other key factors, we need to investigate sociodemographic differences in delayed or interrupted care. Further, the significant economic effects of the COVID-19 pandemic as well as the high rates of job loss may result in the loss of insurance. Thus, more work is needed to understand how these factors affect health care. The potential impacts on patient-level outcomes such as mental health, quality of life, and pain must also be considered.

### Study Limitations

There are some key limitations that need to be considered. First, this study relied on a convenience sample that is not representative in terms of race, ethnicity, education, age, and gender. Convenience samples are limited in terms of the generalizability of prevalence rates. However, they have the advantage of efficiency, which is critical in a dynamic situation, and can be useful for understanding patterns and relationships associated with a phenomenon of interest. Recent research has shown that internet-based convenience samples correlate with truly random probability samples to a surprising extent. For example, Mullinix et al [44] found a correlation of 0.75 when they conducted 20 experiments that replicated results based on national probability samples on Amazon Mechanical Turk (an internet-based survey platform). More recently, Coppock [45] conducted 15 replication experiments and similarly found that convenience and national probability samples provided similar estimates of treatment and moderator effects. However, despite the limitation of using a convenience sample, our study is unique and critical to documenting experiences at the outset of the pandemic, given that the conditions under which the data were collected are not replicable. In addition, a study conducted over the internet does not reach individuals who do not have access to the internet. However, this is perhaps the only mechanism for capturing data from a fleeting moment in time that is characterized by dynamic and uncertain circumstances.

### Conclusions

The pandemic has amplified people’s attention to health disparities and inequities in the United States and has created a need for inclusive and equitable research. The limitations of this study highlight pervasive gaps and challenges associated with conducting such research (and doing so efficiently, given the dynamic nature of the COVID-19 pandemic). There is an urgent need for sharing lessons learned, disseminating effective strategies for reaching more diverse populations (eg, engaging leaders of marginalized communities, understanding and addressing research hesitancy, etc), and encouraging the research community to use and improve upon these strategies in future research. Although the conditions for this study cannot be replicated, the methodological lessons learned can serve as a sort of pilot study for future crises, thereby creating more diverse and inclusive bodies of research that drive health equity forward. Without explicit discussions of research limitations, the research community cannot make progress in collecting data to inform the design of effective programs for addressing health inequities that have existed long before the COVID-19 health crisis. The impact of health care disruptions resulting from the COVID-19 pandemic may be difficult to measure in the short term. However, characterizing these disruptions and improving research methods for such characterizations is critical to informing systemic and equitable planning, mitigation, and recovery strategies for the long term.
